# Canine atopic dermatitis clinical trials: study design and methodological considerations

**DOI:** 10.3389/fvets.2026.1878714

**Published:** 2026-10-09

**Authors:** Michael Klotsman, Sharon K. Sagiv, Wayne H. Anderson, Domenico Santoro

**Affiliations:** 1 Okava Pharmaceuticals, San Francisco, CA, United States; 2 School of Public Health, University of California, Berkeley, Berkeley, CA, United States; 3 Pulmonary and Critical Care Medicine, University of North Carolina, Chapel Hill, NC, United States; 4 University of Florida College of Veterinary Medicine, Gainesville, FL, United States

**Keywords:** Bias (epidemiology), canine atopic dermatitis, clinical trial methodology, dogs, randomized controlled trials, reproducibility of results, treatment outcome, veterinary clinical trials

## Abstract

Canine atopic dermatitis (AD) is a chronic inflammatory and pruritic skin disease affecting up to 15% of dogs, with clinical expression shaped by genetic, environmental, and microbial factors. Although the International Committee for Allergic Diseases of Animals (ICADA) established COSCAD as a core outcome set providing standardized outcome measures for evaluating treatment efficacy, comparatively little attention has been given to the upstream methodological considerations that determine the validity and interpretability of trial results. This narrative review uses a structured methodological framework to examine key sources of random error, bias, confounding, and effect modification that can compromise randomized controlled trials (RCTs) in canine AD. We identify critical parameters requiring careful control in trial design, including food and environmental allergen exposures, seasonality, flea infestation, concurrent bacterial and fungal infections, concomitant therapies, and washout period determination. Historical study protocols, conducted primarily in treatment-naïve dogs with mild-to-moderate disease, are analyzed to illustrate how pivotal registration trials mitigated these sources of error through strict case definitions, medication-specific washout intervals, concomitant therapy restrictions, and rigorous blinding and randomization. We further examine the trade-offs between internal validity and external generalizability, and the growing tension between traditional explanatory trial designs and real-world feasibility in an evolving therapeutic landscape. Synthesizing perspectives from a panel of veterinary dermatology experts, we highlight how disease severity, treatment history, and therapeutic modality should inform eligibility criteria, endpoint selection, and assessment timing. Based on these assessments, we highlight the limitations of applying standardized study design methodologies to such a heterogeneous disease. Applying uniform methodologies to such a heterogeneous syndrome risks obscuring clinically meaningful differences in disease presentation and treatment response. Progress requires flexible, fit-for-purpose study designs that align key methodological elements with the phenotype under investigation, enabling accurate interpretation of treatment effects and more effective therapeutic development.

## Introduction

Canine atopic dermatitis (AD) is a chronic inflammatory and pruritic skin disease that affects up to 15% of all dogs, making it one of the most prevalent and commonly treated conditions in veterinary clinics (1, 2). The clinical manifestation of canine AD encompasses a wide spectrum of presentations, reflecting the complex interplay of various contributing factors (3); clinical expression is shaped by genetic predisposition (e.g., breed-associated phenotypes), lesion distribution (localized versus generalized), stage of the disease (acute flaring *versus* chronic), epidermal-barrier defects, and the presence of cutaneous dysbiosis leading to microbial infections or other flare factors (4–6). Because of this complexity, many dogs require a multimodal treatment approach that includes topical and systemic therapies to provide itch relief and to resolve lesions associated with cutaneous inflammation (7–9).

Four systemic drug classes are currently approved by the FDA for treatment of canine AD. The calcineurin inhibitor ciclosporin (Atopica, Elanco) was the first immunomodulator approved for the treatment of canine AD in 2003, with a ciclosporin oral solutions subsequently approved in 2020 (Cyclavance, Virbac) and 2023 (Modulis, CEVA) (10, 11). More recently, oclacitinib (Apoquel, Zoetis), the first Janus-kinase (JAK) inhibitor for veterinary use, was introduced in 2014 (12). A second JAK inhibitor, ilunocitinib (Zenrelia, Elanco), gained approval in 2024 (13). Most recently, the Committee for Veterinary Medicinal Products (CVMP) adopted a positive opinion for a third highly selective JAK1 inhibitor, atinvicitinib (Numelvi, Merck) in 2025 (14). The first monoclonal antibody (mAb) approved for a canine disease by the United States Department of Agriculture (USDA), the anti-interleukin (IL)-31 lokivetmab (Cytopoint, Zoetis) (15, 16), became available in 2017.

Despite these advances, an estimated 10 to 30% of dogs fail to respond, relapse, and/or experience side effects that necessitate discontinuation of conventional treatments (8, 17–19). The multifactorial nature of AD, characterized by a diverse array of genetic, immunologic, and environmental contributors requires a nuanced and personalized approach to the study of novel treatments and patient subgroups. Consequently, to advance the development of canine AD treatment options and optimize patient care, study designs that capture the heterogeneity of this complex and multifactorial condition are needed.

Clinical practice guidelines and narrative reviews have comprehensively summarized therapeutic options for canine AD and the underlying clinical evidence (7–9, 20). Accordingly, this review does not aim to reiterate treatment recommendations or to provide a systematic review or meta-analysis, but rather to examine how study design choices influence the conduct and interpretation of randomized clinical trials (RCTs) in canine AD. We begin by reviewing the primary endpoints established for canine AD and consider how random and systematic errors can distort observed associations, leading to overestimation, underestimation, or reversal of the true treatment effect. We then analyze historical RCT protocols, conducted primarily in treatment-naïve dogs with mild-to-moderate disease, to illustrate how earlier studies were designed to mitigate these sources of error.

Studies were selected from the peer-reviewed literature, with primary emphasis on published pivotal registration trials supporting regulatory authorization by the FDA, USDA, and/or EMA of contemporary systemic therapies for canine AD, including oclacitinib, lokivetmab, and ilunocitinib (Supplementary Table 1) (15, 16, 19, 21–24). These trials were selected because they provide established regulatory precedents for canine AD trial design. The relevant trials were identified using publicly available Freedom of Information summaries and associated regulatory documentation, and the corresponding peer-reviewed publications were located through targeted PubMed searches. Additional peer-reviewed publications from 2006 through 2025 were identified through supplementary PubMed searches and evaluated for evidence that supported, qualified, or challenged the interpretations and recommendations presented in this review. Because this was a focused narrative rather than a systematic review, the searches were not intended to be exhaustive or to support formal grading of evidence certainty.

Finally, perspectives from seven experts in veterinary dermatology and clinical trial design (see Acknowledgements) were obtained to contextualize methodological trade-offs across disease severities and treatment settings. The experts were selected by the authors based on their published expertise and/or clinical trial experience in canine AD. Input was elicited during a virtual roundtable using a structured discussion guide addressing methodological topics identified by the authors in advance. The authors documented and synthesized the feedback and used it to inform the relevant sections of the manuscript. These perspectives informed the discussion of how study-design approaches may require adaptation to disease severity, treatment setting, and anticipated therapeutic effect. Collectively, they underscore the limitations of a one-size-fits-all approach to trial design in a heterogeneous disease, which may obscure clinically meaningful treatment signals within distinct patient subgroups.

## Endpoints established for canine AD studies

The primary goal of RCTs, the gold standard for determining the effect of therapeutic interventions, is to obtain valid and quantifiable measures of the treatment effect on patient outcomes (25). Well-designed RCTs require the measurement of study endpoints that provide reliable signals of the intervention being tested. In dermatologic disorders such as canine AD, assessing efficacy is challenging because objective laboratory markers of disease severity are lacking (26–28). To address this, the International Committee for Allergic Diseases of Animals (ICADA) established a core outcome set (COS) of subjective but validated instruments to grade the severity of skin lesions and pruritus for canine AD (COSCAD) (29, 30).

The Canine Atopic Dermatitis Extent and Severity Index (CADESI) is a composite scoring index that is used by veterinary dermatologists to systematically score the severity of skin lesions in atopic dogs (31, 32). The Pruritus Visual Analog Scale (PVAS), an owner assessed instrument, is used to evaluate the severity of pruritus (30, 33). Accordingly, these instruments are the most common primary endpoints used in canine AD RCTs (19, 24, 34, 35). To enhance the depth and breadth of a study’s findings, investigators can also include standardized secondary endpoints such as owner global assessment of treatment efficacy (OGATE) and investigator global assessment (2D-IGA). Incorporating these additional measures deepens study insights that can support clinical decision-making and guide future research (29).

## Randomization, random error, bias, confounding and effect modification in canine AD studies

Although validated endpoints have been established for canine AD, random error (or chance) and systematic errors (or systematic bias) need to be minimized to allow for valid and precise inferences between an intervention and outcome(s) to be made (36). Random error, which may be defined as the natural fluctuations that occur in data due to chance, can influence the precision of virtually any outcome measure. Systematic bias, on the other hand, refers to consistent and reproducible errors introduced into the study design, data collection, analysis, and/or reporting that lead to incorrect estimates of the true effect of the intervention. Systematic errors may strengthen, weaken, or completely reverse the true treatment effect (37, 38).

In RCTs, randomization procedures that assign participants to treatment groups by chance increase the likelihood that groups are comparable at baseline by balancing both measured and unmeasured prognostic factors. When implemented correctly, randomization strengthens causal inference by increasing confidence that observed differences in outcomes are attributable to the intervention rather than to confounding variables.

With increasing sample size, randomization more reliably distributes variables associated with the outcome of interest across treatment groups, thereby reducing the risk of chance imbalances. Larger sample sizes also reduce random error, resulting in more precise effect estimates and narrower confidence intervals. However, while randomization improves the probability of balanced groups, perfect comparability cannot be guaranteed in any single trial, particularly when sample sizes are modest. For example, in a randomized controlled trial comparing the oral antihistamine fexofenadine with the active comparator methylprednisolone, baseline imbalances in pruritus scores were observed between treatment groups comprising 15 dogs each (39). Although fexofenadine is not a contemporary standard therapy for canine AD, the study provides a useful methodological example of the potential for chance baseline imbalance in small veterinary trials. Moreover, the wide ranges of baseline CADESI and pruritus scores reported in this study illustrate how substantial within-group heterogeneity can reduce the precision of treatment-effect estimates in small study populations. To mitigate these risks, investigators should incorporate *a priori* sample size and power calculations and consider randomization strategies tailored to study size and design (e.g., blocked or stratified randomization in smaller trials).

Given the limitations of randomization, additional methodological safeguards may be necessary to reduce the impact of systematic error. In canine AD studies, several factors can significantly influence outcome measurements (e.g., skin lesion and pruritus severity scores) and must be carefully considered in trial design and conduct. Known factors that can trigger acute flares and/or change the clinical features of canine AD include exposure to food and environmental allergens, seasonality, and concurrent skin infections and flea infestations (Figure 1). If not appropriately controlled or accounted for, these factors can introduce bias or confounding, potentially masking true treatment effects. The potential impact of these key parameters on canine AD outcome measures, and how they have been addressed in prior studies, is summarized below.

**Figure 1:**
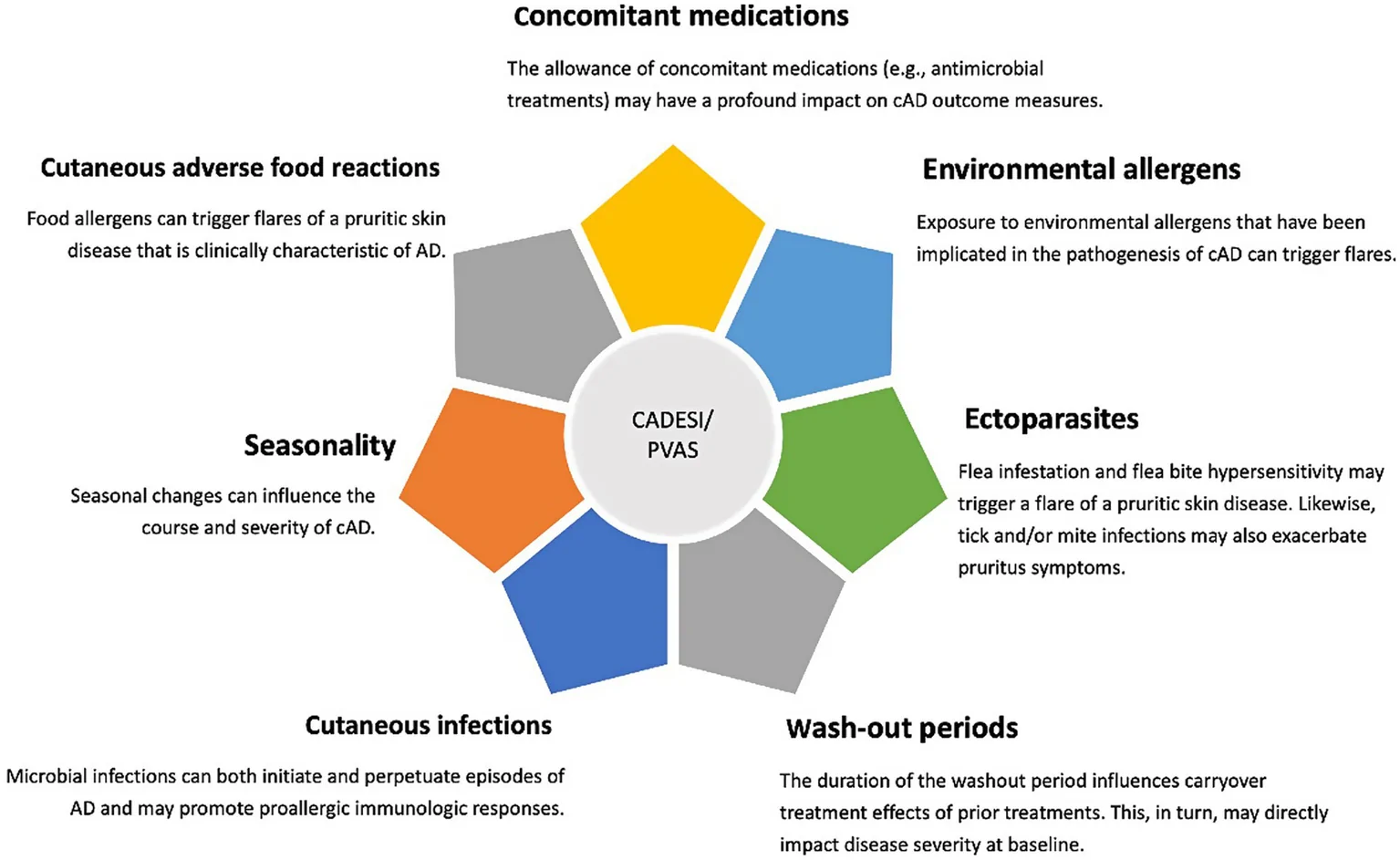
Overview of the principal disease- and treatment-related factors that may influence primary outcome measures (CADESI and PVAS) in randomized clinical trials of canine atopic dermatitis. Further discussion of these factors is provided in the corresponding sections of the main text.

Other sources of bias, well recognized in human clinical research, that may be encountered in veterinary clinical trials include selection bias (systematic differences between animals enrolled in a study and those not enrolled), misclassification bias (systematic differences in how exposures or outcomes are measured across groups), recall bias (systematic errors in the reporting of exposures or events), and attrition bias (systematic differences in withdrawals or exclusions) (Table 1) (40). These categories describe systematic errors that can distort the validity of between-group comparisons.

**Table 1:** Common sources of bias in canine atopic dermatitis clinical trials.

Type of bias	Description	Illustrative example in a canine AD trial
Selection bias	Systematic differences between the characteristics of animals selected for the study and those not selected.	A trial recruiting only from referral dermatology practices may enroll dogs with more severe or treatment-refractory AD compared to the general population of atopic dogs, limiting generalizability of results
Misclassification bias	Systematic differences in how exposures, outcomes, or covariates are measured or classified between groups	In an unblinded trial, investigators aware of treatment allocation may systematically score CADESI-04 lesions more favorably in dogs receiving the investigational therapy than in those receiving placebo, leading to differential outcome assessment and inflation of the apparent treatment effect
Recall bias	Systematic differences in the accuracy or completeness of recalled information between groups	Owners of dogs experiencing more severe or recent atopic dermatitis flares may recall and report prior dietary exposures or environmental triggers differently than owners of dogs with milder disease, potentially creating spurious associations between reported exposures and disease severity
Attrition bias	Systematic differences in withdrawals or exclusions of animals from the study between treatment groups	Dogs experiencing inadequate response or adverse events may be more likely to withdraw from one treatment arm, leading to biased estimates of efficacy if missing data are not handled appropriately. For example, in the oclacitinib registration trial, more than 86% of placebo-treated dogs transitioned from the randomized comparison to an open-label study after Day 28, creating differential attrition that the authors noted biased longer-term between-group comparisons.
Performance bias	Systematic differences in care, management, or co-interventions between treatment groups apart from the intervention under study	Especially when blinding is incomplete, dogs in one treatment arm may receive more frequent follow-up visits, adjunctive topical therapies, or additional owner counseling than dogs in the comparator group, potentially exaggerating observed treatment effects

These sources of bias are also well recognized in randomized controlled trials in human medicine.

The triadic relationship among the animal, owner, and investigator introduces distinctive methodological considerations in veterinary clinical trials, affecting enrollment, treatment administration, outcome assessment, and study retention. This structure has close analogs in proxy-reported outcomes in human pediatric and geriatric research (e.g., parents reporting on behalf of young children (41), caregivers reporting on behalf of patients with cognitive impairment). Firstly, canine AD trials commonly use owner-assessed PVAS together with investigator-assessed CADESI. Discordance between these measures is not inherently evidence of bias because they assess complementary manifestations of disease captured in different settings: owners observe pruritic behavior longitudinally in the home, whereas investigators assess inflammatory skin lesions during clinic visits. However, inconsistency within either measure, variation among raters, or other assessment error unrelated to treatment allocation may introduce nondifferential measurement error. Because such error is not systematically related to treatment assignment, it generally does not favor one group over another; rather, it reduces precision and weakens the ability to detect a true treatment effect and, depending on the outcome scale and error structure, may attenuate observed between-group differences.

Second, a related but distinct phenomenon is the caregiver placebo effect, or “placebo by proxy,” in which an owner’s expectations, treatment preferences, or emotional investment influence the perception and reporting of the animal’s response and may also alter caregiving behavior (42). Caregiver placebo effects have been documented in both owners and veterinarians, including under randomized, double-masked, placebo-controlled conditions (43). Expectancy effects may increase apparent improvement across treatment groups, including among placebo recipients, and can thereby reduce assay sensitivity; when comparable between groups, they do not systematically favor either group. If expectations or perceived treatment allocation differ between groups, however, they may introduce systematic observer bias and distort the estimated treatment effect.

Finally, owner-related factors may influence enrollment. Because owners determine whether their animals participate, owner motivation, treatment preferences, available resources, schedule flexibility, and willingness to comply with study procedures may shape the enrolled population. Recruitment exclusively through referral dermatology practices may overrepresent dogs with more severe or treatment-refractory AD, while protocols requiring frequent visits, extensive diagnostic testing, or elimination diets may preferentially enroll owners with greater resources or treatment motivation. This owner-mediated participation effect may limit the representativeness and generalizability of the study population, even when randomization adequately balances the enrolled treatment groups.

These sources of bias and variability are particularly consequential in canine AD trials because of modest sample sizes, reliance on owner-reported outcomes, disease heterogeneity, and variability in clinical care. Potential mitigation measures include masking; validated, complementary owner- and investigator-assessed outcomes; standardized assessor training and investigator–owner communication; completion of owner assessments before consultation with the investigator; contemporaneous electronic diaries; use of the same owner-reporter throughout the study when feasible; adherence monitoring; and prospective documentation of concomitant treatment, rescue therapy, and reasons for withdrawal.

Confounders and effect modifiers are both variables that can influence study outcomes, but they do so in distinct ways. A confounder is a variable associated with both the exposure (e.g., interventional treatment for canine AD) and the outcome (e.g., CADESI), and can distort the observed relationship between them. For example, in a study evaluating a new drug for canine AD, flea insecticidal use could act as a confounder if dogs with flea infestation (FI) are more likely to exhibit AD symptoms and are also more likely to receive the intervention. If not appropriately controlled, this could lead to a spurious association between the treatment and clinical outcomes.

In contrast, an effect modifier alters the strength or direction of the association between treatment and outcome across its levels. Rather than introducing bias, effect modification reflects true heterogeneity in treatment response. In the same example, breed could function as an effect modifier if differences in pharmacokinetics or metabolism influence drug response across breeds (44). More broadly, clinically or immunologically defined phenotypes within the heterogeneous AD syndrome may function as effect modifiers if treatment response varies across subgroups. Whether a given phenotypic distinction should inform case definition, stratification, or prespecified subgroup analysis depends on the intervention and trial objective.

## Environmental exposures associated with canine AD endpoints

### Food allergens

Food allergens are a common cause of dermatologic disease in dogs and can exacerbate AD; they may trigger a flare of a pruritic skin disease that is clinically characteristic and indistinguishable from AD (45–47). Consequently, enrolling dogs with undiagnosed or uncontrolled food allergy can compromise the precision and accuracy of trials designed to evaluate AD interventions. To minimize potential food allergy effects in explanatory efficacy trials, eligibility often requires an investigator-determined diagnostic evaluation sufficient to rule out food allergy, with elimination-diet testing undertaken when warranted by the dog’s clinical history and prior diagnostic work-up (Supplementary Table 1). Once enrolled, all dogs should remain on a consistent diet for the duration of the study to prevent diet-induced flares from distorting the true treatment effects.

### Environmental allergens

Numerous environmental allergens, including pollens from grasses, trees, and weeds, as well as insect, mold, mite, and microbial allergens, contribute to the pathogenesis of canine AD (47, 48). Complete avoidance is not feasible; exposure varies by geography and season and may precipitate disease flares. Because imbalances in environmental allergen exposure across groups can obscure true treatment effects (49), well-designed clinical trials aim to minimize and account for exposure-related heterogeneity where feasible. This may include restricting enrollment by season or geography, standardizing background care (e.g., flea control), and using blocked or stratified randomization by site or baseline disease severity. In smaller studies, additional design and analytic strategies (e.g., limiting the number of sites and prespecifying covariate adjustment for site, season, or exposure proxies) may help reduce residual variability, while recognizing that complete control of environmental exposure is not achievable.

### Seasonal atopic dermatitis

Seasonal changes can influence the assessment of disease severity in canine AD (24, 50, 51). As such, seasonality may affect the magnitude or direction of observed treatment effects (e.g., dogs enrolled during low-allergen winter months may respond differently than dogs enrolled during peak pollen seasons), particularly if enrollment is imbalanced across seasons between comparison groups. In longer-duration studies (e.g., >90 days), underlying seasonal variation may further complicate interpretation of treatment response over time.

To mitigate these effects, many interventional randomized controlled trials restrict enrollment to dogs with non-seasonal AD, defined as dogs with year-round clinical signs, while recognizing that such dogs may still experience seasonal exacerbations. Accordingly, even within non-seasonal AD populations, careful consideration of enrollment timing, study duration, and randomization is required to minimize seasonal imbalances and their potential impact on outcome measures.

## Accounting for flare factors of itch

### Ectoparasites

In dogs, flea infestation (FI), flea bite hypersensitivity (FBH), and other ectoparasitic diseases such as sarcoptic mange may cause pruritus that exacerbates or mimics the clinical signs of AD. To control for these confounders, enrollment should be restricted to dogs with no clinical signs suggestive of FI, FBH, or other active ectoparasitic disease (52). As detailed below, ectoparasite-control regimens should be maintained throughout the entire RCT.

### Bacterial/fungal infections

Dogs with canine AD frequently present with concurrent bacterial and/or fungal (yeast) infections (53, 54). Careful consideration of infection status at baseline (i.e., overgrowth *versus* active infection, severity, and distribution) as a study entry criterion is warranted, because cutaneous infections can promote pruritus and induce or exacerbate inflammation, thereby worsening the clinical signs of AD. To minimize the impact infections may have on canine atopic dermatitis outcome measures, dogs with active bacterial folliculitis or *Malassezia* dermatitis are often excluded at study entry from interventional AD trials (19, 34, 55). This approach reduces confounding by infection-related pruritus and inflammation, allowing for clearer interpretation of treatment effects attributable to the investigational therapy.

No validated, universally accepted quantitative thresholds alone distinguish clinically active bacterial or Malassezia dermatitis or otitis from microbial overgrowth or colonization in dogs with cAD (56). Accordingly, trial protocols should prespecify operational definitions and standardized assessment procedures appropriate to the study objective rather than rely on an unspecified designation of “active infection.” In practice, assessment may combine compatible clinical signs, lesion morphology and distribution, otoscopic findings, and cytology obtained from prespecified skin and, where relevant, otic sites, with bacterial culture and antimicrobial susceptibility testing performed when clinically indicated. Because no single quantitative threshold or diagnostic algorithm is universally accepted, protocols should specify the criteria used to determine infection status, the sites and timing of sampling, cytologic methods and decision rules, investigator training, and criteria for infection resolution or stabilization before randomization. In explanatory efficacy trials, clinically active infection may be treated and stabilized before randomization to reduce confounding of pruritus and lesion endpoints. In trials enrolling infection-prone or otherwise complex canine AD populations, susceptibility to recurrent infection may instead be retained as part of the clinical phenotype and prospectively characterized as described below (see “Multimodal Treatments and Supplemental Endpoint Measures”).

## Impact of concomitant treatments on canine AD symptoms

### Antibacterial and antifungal therapies

In dogs with AD and concurrent cutaneous infections, antibacterial and antifungal therapy may lead to partial or marked reductions in clinical signs (53). Accordingly, the allowance and timing of concomitant antimicrobial therapies can substantially influence AD outcome measures. To minimize confounding from infection-related pruritus and inflammation, many interventional trials exclude dogs with active infections at enrollment and require an appropriate washout period (discussed below) prior to baseline assessment.

Alternatively, some studies permit concomitant antimicrobial therapy to better reflect real-world clinical practice. In such designs, the use, timing, and duration of these therapies should be documented and may be reported as a secondary or rescue-treatment endpoint. Interpretation of efficacy should account for whether the study is intended to isolate AD-specific treatment effects or to evaluate treatment performance within a multimodal, pragmatic clinical context.

### Ectoparasite control

To minimize the risk of ectoparasite-related flares, including those associated with FI, FBH, and pruritic tick and mite infestations such as sarcoptic mange, an effective ectoparasite-control regimen should be maintained throughout the study period. The specific product used may vary according to geographic location and local parasite burden; however, the protocol should define minimum coverage and administration requirements, and control should remain consistent and uninterrupted for all enrolled dogs throughout the study, regardless of site. Where pertinent, use of agents that also provide acaricidal activity may further reduce the risk of mite-associated pruritic flares. Likewise, interventions aimed at reducing exposure to or harmful sequelae from environmental triggers of AD could prevent or mitigate disease.

### Washout period

Washout periods are defined as the interval during which prior treatments are discontinued and cleared from a patient’s system before baseline assessment. Their purpose is to minimize carryover effects that could confound interpretation of treatment response. The required duration varies by medication; in general, longer washouts are needed for systemic therapies compared with topical agents, and for depot versus non-depot formulations.

Published canine AD protocols illustrate how washout requirements may vary according to treatment class, formulation, and route of administration. For example, lokivetmab was subject to an eight-week washout in the ilunocitinib registration trial (22). The underlying carry-over concern is clinically plausible because lokivetmab can produce antipruritic effects lasting several weeks after administration (57, 58); however, the available data do not establish 8 weeks as a universally validated washout threshold. Glucocorticoid protocols have similarly distinguished between long-acting injectable formulations (e.g., methylprednisolone acetate), requiring washouts of six to 8 weeks, and shorter-acting formulations (e.g., methylprednisolone succinate), requiring approximately 4 weeks. In FDA registration trials for canine AD, washout periods ranged from 1 week for oral antibacterial and antifungal agents to 4 weeks for long-acting injectable antimicrobials (21, 34). Certain topical therapies have been assigned washouts of up to 14 days. More broadly, these intervals should be regarded as protocol precedents rather than universally validated thresholds. Washout selection should account for the treatment’s formulation and route of administration, resolution of any underlying infection, and potential residual effects on the selected clinical outcome measures. Pharmacokinetic elimination, target engagement and pharmacodynamic duration, and the duration of measurable clinical benefit are related but distinct considerations. When drug-specific evidence is unavailable, elimination half-life may provide a preliminary reference but should not be used in isolation (59), while recommended medication-withdrawal intervals for intradermal allergy testing may offer additional practical guidance (60).

Incorporating washout periods in canine AD trials is not straightforward. Although longer washouts reduce the risk of carryover effects, they may also exacerbate clinical signs as disease remains untreated. This challenge is compounded for systemic therapies with delayed onset or prolonged biological effects, where fixed time-based washouts may not fully capture residual activity. As a result, extended washout requirements can introduce ethical and practical constraints, particularly in dogs with more severe disease, by limiting recruitment and reducing owner willingness to enroll.

Depending on the trial objective, alternative designs may reduce reliance on uniform, lengthy washout periods that may be impractical to implement. Alternative options include trials conducted with standardized stable background therapy; add-on, steroid-sparing, or antimicrobial-sparing designs (see “Multimodal Treatments and Supplemental Endpoint Measures”); active-comparator trials in which the study intervention is compared with an approved therapy rather than placebo; and randomized-withdrawal designs. Prespecified rescue-treatment algorithms may also be incorporated, with rescue use addressed prospectively in the definition and analysis of the treatment effect (see “Multimodal Treatments and Supplemental Endpoint Measures”).

## Study design conventions for frontline canine AD treatment options

### Case-study

The pivotal oclacitinib (Apoquel®, Zoetis) registration trial illustrates key study design methodologies for evaluating frontline canine AD treatments (34). The subsequent ilunocitinib (Zenrelia™, Elanco) registration trial largely retained the design framework established in the oclacitinib trial: a multicenter, randomized, double-masked, placebo-controlled design with primary efficacy assessed on Day 28 (22). Although the protocols differed in certain operational features, both applied the same core methodological principles, including defined diagnostic and eligibility criteria, medication-specific washout periods, restrictions on concomitant therapies, random treatment allocation, and masking. We therefore examine the oclacitinib protocol in detail to demonstrate how these design elements were used to mitigate potential sources of bias and minimize error in endpoint assessment.

### Case-definition

To maximize internal validity, the oclacitinib registration study applied the Prélaud criteria as a prespecified clinical case definition for canine AD, a diagnostic framework that preceded the prospectively derived Favrot criteria (34, 54, 61). Irrespective of the specific clinical criteria used, consistent application of a prespecified case definition promotes standardized enrollment and supports internal validity; however, differences in the component criteria and diagnostic performance of the selected framework may influence case classification, the composition of the enrolled population, and the generalizability of the findings.

To further minimize the influence of environmental exposures and flare triggers associated with AD phenotypes, investigators excluded dogs with food allergies, FBH, bacterial or *Malassezia* dermatitis and/or otitis, internal and external parasitism, metabolic disease, and other conditions. The recruitment of tightly screened cases minimized statistical noise from extraneous flare triggers, thus strengthening the causal link between treatment and outcome. However, the trade-off for enhancing internal validity by recruiting a homogeneous subset of AD cases is a reduction in external validity. The findings may be generalized to dogs with comparable clinical profiles but may be less applicable to other AD phenotypes or more complex clinical presentations. In trials intended to enroll broader or more complex populations, concurrent bacterial or Malassezia dermatitis or otitis need not be categorically excluded but should be assessed and managed according to prespecified criteria appropriate to the trial objective (see “Bacterial/Fungal Infections”).

### Carry-over effect

To ensure that improvements in CADESI-02 and PVAS were attributable to oclacitinib rather than lingering effects of previous drugs, the protocol imposed medication-specific washout intervals. Wash-out durations were matched to each agent’s pharmacodynamics: 6 weeks for depot glucocorticoids; 4 weeks for oral glucocorticoids, ciclosporin, long-acting injectable antimicrobials, and other miscellaneous compounds with known antipruritic activity, including Staphage Lysate (SPL; Delmont Laboratories Inc., Swarthmore, PA, United States), gabapentin, monoamine oxidase inhibitors, and tacrolimus; 3 weeks for topical NSAIDs or topical steroids; 2 weeks for antihistamines; and 1 week for systemic antibacterial or antifungal agents. These wash-outs minimized carry-over bias, ensuring that clinical responses were attributable solely to the study treatment and not to residual effects from prior interventions. In practice, however, lengthy washouts can be impractical or even unethical. Owners may be reluctant to withhold relief from severely pruritic dogs, while investigators may struggle to recruit animals as the standard of care evolves and the pool of eligible patients shrinks.

### Concomitant therapy

To minimize concomitant treatments from confounding CADESI-02 and PVAS outcome measurements, the protocol offered two options: discontinue the treatment with a corresponding washout period or continue their use under predefined *minimal-use/minimal-frequency* rules [see Cosgrove et al., Supplementary Table 1 (34)]. Importantly, medicated shampoos and all systemic or topical antimicrobials (i.e., interventions with antipruritic activity), were prohibited until after the Day 28 primary endpoint assessment.

Dogs receiving therapy for unrelated chronic disorders (e.g., hypothyroidism, osteoarthritis) could enroll only if the regimen had been unchanged for at least 6 weeks before Day 0 and it was not expected to change during the 28-day study. This requirement further reduced the risk that shifts in concurrent medication would bias lesion- or pruritus-based endpoints.

### Blinding and randomization

Finally, the trial was designed as a prospective, randomized, double-blind, placebo-controlled study. Blinding kept owners, investigators, and data assessors unaware of treatment allocation. Blinding helps minimize the influence of treatment expectations, which is particularly important when outcomes such as investigator-assessed CADESI and owner-assessed PVAS are subjective (62).

Randomization assigns dogs to treatment or placebo purely by chance; with adequate sample size, this process balances both known and unknown prognostic factors across groups, minimizing selection bias and supporting valid statistical inference. Together, randomization and blinding enhance the internal validity of the study and bolster confidence that any differences in outcomes reflect the effect of oclacitinib rather than spurious associations secondary to random or systematic errors.

## Clinical trial design and accounting for canine atopic dermatitis heterogeneity

Well-designed canine AD trials, exemplified by the oclacitinib and ilunocitinib registration studies, incorporate several core study design elements (Table 2). Protocols should: (i) *a priori* specify validated endpoint measures (e.g., CADESI and PVAS); (ii) employ rigorous eligibility criteria to ensure the study population has a true AD diagnosis; and (iii) account for parameters that may directly influence canine AD (e.g., mandate flea treatment to control for FI and FBH). However, while these core principles are essential, a standardized, one-size-fits-all approach is insufficient, as it may fail to address the clinically significant variations across different patient populations and treatment goals.

**Table 2:** Suggested design considerations for randomized clinical trials in canine atopic dermatitis.

Trial-design component	Recommendation
Case definition and eligibility	Apply a validated, prespecified case definition (e.g., Favrot criteria). Exclude relevant differential diagnoses and prespecify how food-induced AD and potentially confounding comorbidities, including flea bite hypersensitivity, ectoparasite infestation, and bacterial or *Malassezia* infection, will be excluded, treated, stabilized, or otherwise accounted for at study entry.
Ectoparasite control	Exclude active ectoparasite infestation at study entry and maintain a consistent, protocol-defined ectoparasite-control regimen throughout the trial. If products vary among sites, prespecify minimum coverage and dosing requirements.
Washout period	Prespecify medication-specific washout periods based on formulation, route of administration, pharmacokinetic and pharmacodynamic properties, and potential residual effects on clinical outcomes. For antimicrobial therapies, also consider whether the treated infection has clinically resolved before baseline. Regard published intervals as protocol precedents rather than universally validated thresholds.
Concomitant medications	Prespecify which therapies are prohibited, permitted, or standardized. Record and report their use, timing, and duration, and consider rescue-treatment use as a supportive outcome.
Randomization and blinding	Use an adequately powered sample and an appropriate randomization scheme; consider blocked or stratified allocation in smaller trials. Maintain masking of owners, investigators, and outcome assessors whenever feasible.
Endpoint selection	Consistent with COSCAD recommendations, include both owner-assessed pruritus (PVAS) and veterinarian-assessed skin-lesion severity (CADESI). Within this framework, select the primary endpoint based on the intervention’s intended effect and mechanism (e.g., PVAS for a pruritus-targeted agent), and retain complementary COSCAD measures as secondary or supportive endpoints to characterize effects across other clinically relevant disease domains.
Assessment timing	Consistent with the clinical goal of rapid symptom control, Day 14 or Day 28 may be appropriate primary efficacy time points for trials of frontline treatments in uncomplicated mild-to-moderate AD. For moderate-to-severe, refractory, or otherwise complex AD, a later primary or key efficacy assessment beyond Day 28 may be warranted to account for greater disease complexity and capture delayed or evolving treatment responses.
Owner–investigator and proxy-reporting effects	Use validated, complementary owner- and investigator-assessed instruments. Standardize instructions and assessor training, obtain owner assessments before investigator consultation, use contemporaneous diaries, and retain the same owner-reporter whenever feasible.

Given the multifactorial and immunologically complex nature of canine AD, trial methodologies must be adaptable to account for disease heterogeneity and the specific mechanism of action of the intervention under study. In designing a protocol, careful consideration should be given to factors such as a dog’s treatment history (i.e., treatment-naïve dogs with no prior exposure to AD treatment(s) versus refractory dogs with minimal or no therapeutic response to prior treatment), severity of disease (i.e., mild-to-moderate versus moderate-to-severe), timing of treatment goals i.e., immediate symptom relief versus sustained efficacy and remission maintenance, and the specific target of intervention (i.e., itch relief versus reduction of cutaneous inflammation versus restoration of skin barrier function). Failure to account for these factors may result in misleading conclusions and may risk mischaracterizing the therapeutic intervention under study.

To illustrate how disease severity and treatment history influence trial design, we highlight how the methodology for a frontline treatment differs from that for second-line therapies in treatment-refractory cases.

### Disease severity, therapeutic modality, and time point of primary endpoint measurement

The time required to assess study endpoints is influenced by disease severity, which shapes treatment goals and expectations. In registration studies of mild-to-moderate canine AD, RCTs often prioritize rapid symptom relief, with endpoints evaluated at earlier timepoints (Figure 2). In contrast, the focus shifts to longer-term disease management in studies designed to evaluate treatment response in severe or refractory cases where dogs may fail to respond to standard treatments, may require a multimodal treatment approach, and/or experience relapses. In studies designed for more complex, severe AD cases which often present with recurrent microbial skin infections, primary endpoint assessment at Day 28 alone may be insufficient to differentiate symptom improvements associated with antimicrobial products from improvements attributed to the intervention under study. We recommend that investigators consider extending the primary or key assessment window beyond Day 28 (e.g., to Day 56 or Day 84, as illustrated in Figure 2) in these populations. These time points are illustrative rather than prescriptive; the timing of the primary endpoint should be informed by the anticipated onset and duration of the intervention’s effect, the study population, and the management of concomitant therapies.

**Figure 2:**
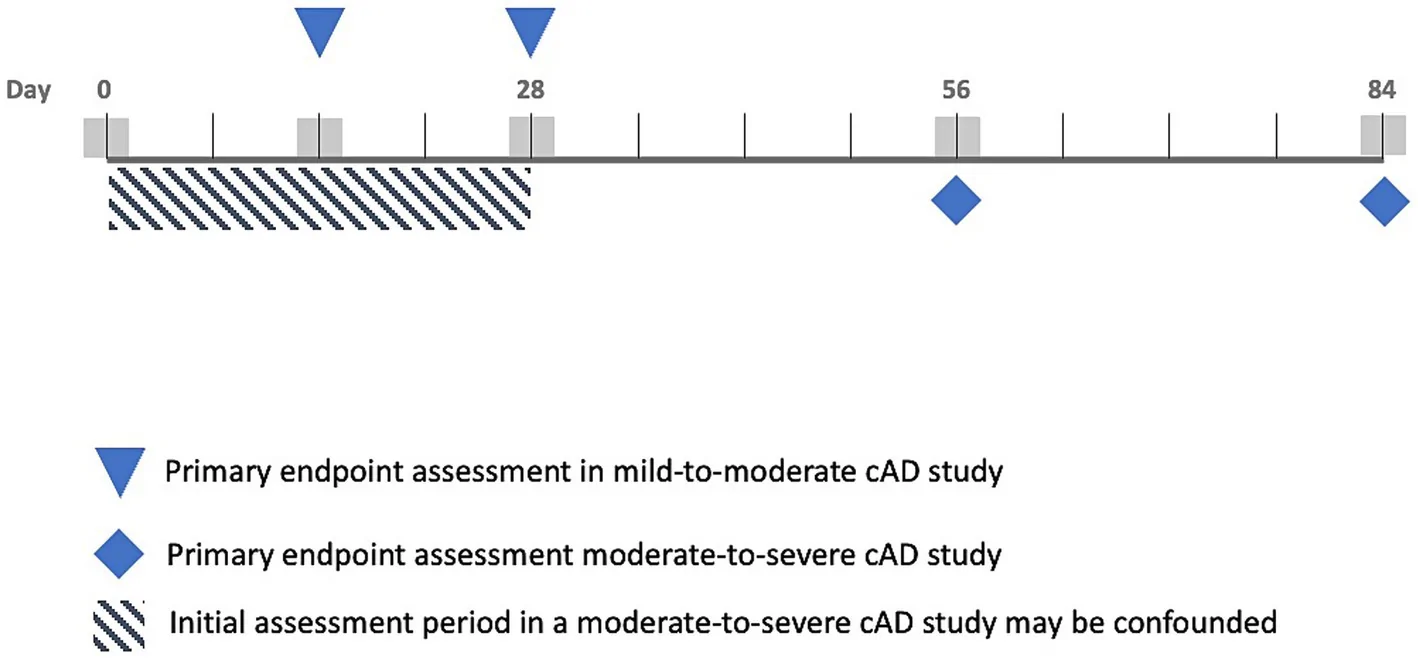
Illustrative study design schema for canine atopic dermatitis (AD) clinical trials. Dogs are randomized on Day 0 with grey boxes representing clinic visits. In clinical trials conducted in mild-to-moderate canine AD, the purpose of the study is to determine whether the treatment effect provides immediate symptom relief. As such, the primary endpoint(s) are evaluated at Day 14 (possibly on Day 7) and/or Day 28. In contrast, a Day 28 assessment in dogs with moderate-to-severe canine AD may be insufficient because of recurrent infections or a delayed or evolving treatment response. A later primary endpoint (e.g., Day 56 or Day 84) may better characterize sustained disease control. These time points are shown as illustrative examples rather than validated benchmarks; the specific timing should be adapted to the clinical context and the anticipated onset, duration, and mechanism of the intervention’s effect.

The time point assessment of the study endpoint(s) should also align with the intended clinical benefit of the intervention and whether the corresponding study goal is to demonstrate immediate symptom relief or long-term management of more complex cases (Figure 2). For example, interventions like ciclosporin, which typically require 4–6 weeks to show clinical effects (9), may necessitate longer study durations. Conversely, shorter assessment periods may be suitable for treatments like corticosteroids or JAK inhibitors, which offer rapid relief of pruritus and inflammation.

### Therapeutic modality and endpoint selection

In determining the primary endpoint(s) for clinical trials in canine AD, it is imperative to align these measures with the therapeutic objectives of the intervention being evaluated. For instance, the advent of emerging therapies specifically targeting itch receptors or neural pathways holds promise in addressing the profound pruritus associated with canine AD. Recent translational research suggests that agents targeting IL-31 inhibition effectively mitigate pruritus while exerting minimal impact on local skin inflammation (57, 63). Consequently, designating a pruritus endpoint (e.g., PVAS), rather than a skin-lesion endpoint (e.g., CADESI), as the primary outcome may be warranted for pruritus-targeted agents, allowing efficacy to be assessed using the outcome most closely aligned with the intervention’s mechanism and intended use. Importantly, other recommended COSCAD outcome measures, particularly veterinarian-assessed skin-lesion severity (e.g., CADESI), should generally be retained as secondary or supportive endpoints to characterize effects across other clinically relevant domains of AD and to avoid interpreting improvement in pruritus as broader disease control.

### Multimodal treatments and supplemental endpoint measures

In clinical scenarios necessitating multimodal treatment approaches, endpoints that complement established endpoints (e.g., CADESI and PVAS) should be considered. For example, studies have shown that short concomitant glucocorticoid administration is beneficial when initiating treatment with ciclosporin or oclacitinib (35, 64). In a ciclosporin study, coadministration of glucocorticoids accelerated reductions in pruritus and CADESI scores. In a separate oclacitinib study, concomitant glucocorticoid therapy reduced the likelihood of rebound pruritus. Treatment sparing (e.g., “steroid sparing”) benefits and an emphasis on safety parameters should be key measures for such multimodal treatment approaches. Likewise, in studies enrolling dogs that are likely to present with concurrent cutaneous infections, metrics capturing drug-sparing effects of antibiotics or antiseptics should also be considered as indicators of treatment success.

Rescue-medication use and discontinuation of randomized treatment are post-randomization events that can affect the interpretation of subsequent outcomes (65, 66). Protocols and statistical analysis plans should prospectively specify how these events will be defined, recorded, and incorporated into the treatment effect of interest and corresponding analysis. Withdrawal from study follow-up may be particularly informative in canine AD trials because owners may withdraw dogs whose clinical signs remain inadequately controlled to avoid prolonging their discomfort. The potential consequences are illustrated by the differential withdrawal observed in the oclacitinib registration trial (Table 1, attrition bias example); such withdrawal may bias treatment-effect estimates and should be anticipated through prospectively defined rescue criteria, continued outcome collection when feasible, and sensitivity analyses addressing informative dropout.

In explanatory efficacy trials, clinically active secondary infection may be treated and stabilized before randomization to reduce confounding of pruritus and skin-lesion endpoints. By contrast, trials enrolling infection-prone or otherwise complex canine AD populations may retain susceptibility to recurrent infection as part of the clinical phenotype rather than excluding it. In these trials, baseline history of recurrent infection and antimicrobial use should be documented and may, as appropriate, be incorporated as stratification factors or potential effect modifiers. Incident infections, antimicrobial exposure, and rescue-treatment use should be prospectively captured as outcomes or post-randomization events affecting treatment-effect interpretation, consistent with the treatment of rescue medication described above. Where antimicrobial or antiseptic therapy is permitted, protocols should prespecify treatment algorithms, including agent selection, route, dose, and duration; criteria for resolution or stabilization and their timing relative to endpoint assessment; and procedures for documenting treatment exposure and adherence.

### Theoretical study design, evolving standard-of-care, and real-world feasibility

In designing canine AD clinical trials, it is essential to consider real-world practicalities, as the ideal theoretical approach may not always be feasible in everyday clinical settings. While certain methodologies or protocols may appear optimal on paper, factors such as the ability to identify and recruit subjects, client compliance, ethical considerations, resource availability, and budget and logistical constraints often shape the success of studies.

Historical trial designs can offer valuable precedents; however, study designs must remain adaptable to evolving standards of care and advances in outcome measurement. For example, following publication of the COSCAD’18 core outcome set, the 0–10 verbal numeric scale was validated for assessing pruritus in dogs (30). Selection of a specific instrument should remain informed by current expert consensus, the relevant instrument-validation literature, and the objectives of the individual trial. Accordingly, different instruments intended to measure the same outcome domain, such as pruritus, should not be assumed to be interchangeable. The contrasting recruitment experiences of the oclacitinib and ilunocitinib registration trials illustrate the potential implications of an evolving clinical landscape. Although these trials differed in certain operational features (Supplementary Table 1), they employed similar placebo-controlled design frameworks, used broadly comparable eligibility criteria to enroll dogs with canine AD, and involved a similar number of participating clinics. Despite these similarities, the oclacitinib study was completed in under 6 months, from August to November 2009, whereas the ilunocitinib study took more than 3 years, from May 2020 to June 2023 (13, 67). This disparity suggests that the expanding availability of effective canine AD treatments may have created practical and ethical barriers to timely recruitment under real-world conditions, particularly for placebo-controlled trials.

As therapeutic options advance, study protocols need to be modernized to ensure participants are not withheld effective treatments or exposed to interventions that are known to be inferior or less effective than available alternatives. This approach allows studies to meet evolving client expectations and avoid ethical dilemmas. Reporting of individual trials should likewise keep pace with current standards, such as those outlined in the PetSORT consensus statement developed specifically for trials in client- and shelter-owned dogs and cats, so that design decisions are described transparently (68, 69).

## Conclusion

Canine AD is a heterogeneous syndrome that presents a multifaceted clinical challenge (3–6), and requires a tailored approach to treatment that encompasses both topical and systemic therapies (7, 8). The array of systemic antipruritic and anti-inflammatory medications commonly employed in dogs, including JAK inhibitors, lokivetmab, ciclosporin, and glucocorticoids, is equally diverse; these agents vary in their mode of action, time-to-onset, and rates of adverse reactions (8, 9). Underscoring the need for individualized treatment strategies that account for factors such as secondary infections, acute inflammatory flares, patient variability, and client compliance and financial considerations, the 2023 American Animal Hospital Association guidelines emphasize that “treating the allergic dog is not one-size-fits-all” (9). By extension, there is no one-size-fits-all methodology for conducting RCTs in canine AD.

Applying uniform trial methodologies risks overlooking clinically meaningful differences in disease presentation, severity, and treatment response. Standard endpoints may also inadequately capture the broader burden of disease on canine AD patients and their owners, particularly in subgroups with comorbid conditions such as recurrent skin infections. Progress therefore requires flexible study designs that align eligibility criteria, washout rules, and outcome measures with the phenotype under investigation, supported by adaptable protocols and patient-centered endpoints that better reflect real-world heterogeneity. Through such approaches, coupled with ongoing translational research, the veterinary community can accelerate the development of effective therapies that address the diverse needs of the canine AD patient population.
