# Supplementary material for: Canine atopic dermatitis clinical trials: study design and methodological considerations
Source: Front Vet Sci. 2026 Oct 9;13:1878714. doi: 10.3389/fvets.2026.1878714 (PMC13692925; doi:10.3389/fvets.2026.1878714)
Supplement: Supplementary file 1 [file Data_Sheet_1.pdf]

S1. Methodological characteristics of randomized field studies and regulatory evidence for systemic therapies in canine atopic dermatitis

Drug / comparison	Reference	Publication / assessment date	Study design and sample	Case definition, eligibility and disease controls	Washout and concomitant therapy	Endpoints, assessment timing and raters
**Oclacitinib vs placebo**	Cosgrove et al., Vet Dermatol. 2013;24:587–597, e141–e142. doi:10.1111/vde.12088<br><br>FDA FOI Summary, NADA 141-345 (AD Studies A16/A17)<br><br>EMA Apoquel EPAR	2013<br><br>FDA approval: 14 May 2013<br><br>EMA assessment: 2013	**Design:** Multicenter, randomized, double-masked, placebo-controlled field trial; 112 days.<br>**Sample/allocation:** 299 client-owned dogs at 18 dermatology specialty clinics; 152 oclacitinib and 147 placebo (1:1, blocked within site).<br>**Masking:** Owners and clinical investigators masked; treatment dispenser unmasked.	**Case definition:** Chronic nonseasonal cAD diagnosed using Prélaud criteria; ≥12 months old; owner-rated moderate-to-severe pruritus; CADESI-02 ≥25.<br>**Controls:** Food allergy, flea-bite hypersensitivity, ectoparasites, bacterial or Malassezia dermatitis/otitis, metabolic disease, and other confounders excluded; dogs flea-free with prevention maintained; diet and allergen-specific immunotherapy stabilized.	**Washout:** Drug/formulation-specific intervals: 1 week for oral antimicrobials; 2 weeks antihistamines; 3 weeks topical anti-inflammatory drugs; 4 weeks ciclosporin, oral glucocorticoids, long-acting antimicrobials and other antipruritics; 6 weeks long-acting injectable glucocorticoids.<br>**Concomitant therapy:** Antimicrobials permitted after Day 28; corticosteroids, antihistamines, ciclosporin and other immunosuppressants prohibited.	**Primary assessment:** Day 28 treatment success, assessed separately for owner PVAS (≥2-cm reduction) and investigator CADESI-02 (≥50% reduction).<br>**Timing/rater:** PVAS on Days 0, 1, 2, 7, 14, 28, 56, 84 and 112; CADESI-02 on Days 0, 14, 28, 56, 84 and 112.<br>**Methodological note:** Most placebo dogs left the randomized comparison by Day 28, limiting longer-term between-group inference.
**Oclacitinib vs ciclosporin**	Little et al., Vet Dermatol. 2015;26:23–30, e7–e8. doi:10.1111/vde.12186	2015	**Design:** Multicenter, randomized, positive-controlled, investigator-masked noninferiority trial; 84 days.<br>**Sample/allocation:** 226 client-owned dogs at eight Australian sites; 114 oclacitinib and 112 ciclosporin (1:1 randomized complete blocks).<br>**Masking:** Investigators masked; owners unmasked because dosing regimens and packaging differed.	**Case definition:** Chronic nonseasonal cAD; ≥12 months old; owner-rated moderate-to-severe pruritus; CADESI-02 ≥25.<br>**Controls:** Screening and stabilization procedures broadly followed the placebo-controlled oclacitinib protocol, including exclusion/control of relevant flare triggers and maintenance of flea control.	**Washout/concomitant therapy:** Protocol-defined withdrawal of potentially interfering therapies; systemic azole antifungals and other prohibited antipruritic or immunomodulatory therapies were not allowed. Permitted treatments were documented.	**Co-primary endpoints:** Percentage reduction from baseline in owner PVAS and investigator CADESI-02 at Day 28; noninferiority margin 15%.<br>**Timing/rater:** PVAS on Days 1, 2, 7, 14, 28, 56 and 84; CADESI-02 on Days 14, 28, 56 and 84. Owner and investigator response-to-treatment assessments were supportive.
**Lokivetmab vs placebo (dose determination)**	Michels et al., Vet Dermatol. 2016;27:478–e129. doi:10.1111/vde.12376<br><br>EMA Cytopoint EPAR	2016<br><br>EMA assessment adopted: 16 Feb 2017	**Design:** Multicenter, randomized, double-masked, placebo-controlled dose-determination field trial; single injection with 56-day follow-up.<br>**Sample/allocation:** 211 client-owned dogs at 15 referral clinics; placebo or lokivetmab 0.125, 0.5 or 2.0 mg/kg subcutaneously (approximately 50 dogs/group).	**Case definition:** Chronic nonseasonal cAD diagnosed using established criteria; ≥12 months old; PVAS ≥3 cm; CADESI-03 ≥30.<br>**Controls:** Food allergy, flea allergy, ectoparasites, bacterial/fungal dermatitis or otitis, metabolic disease and other relevant differentials excluded; dogs flea-free with prevention maintained; diet and immunotherapy stabilized.	**Washout:** 1 week oral antimicrobials/topical anesthetics; 2 weeks antihistamines; 3 weeks topical anti-inflammatory drugs; 4 weeks ciclosporin, oral glucocorticoids, long-acting antimicrobials and other antipruritics; 6 weeks long-acting corticosteroids; 8 weeks oclacitinib.<br>**Concomitant therapy:** Potentially outcome-altering therapies restricted; specified stable therapies permitted.	**Primary efficacy variables:** Regulatory analysis of treatment success using owner PVAS (≥2-cm reduction) and investigator CADESI-03 (≥50% reduction) at Days 28, 42 and 56; dose response and duration were central objectives.<br>**Timing/rater:** PVAS frequently through Day 56; CADESI-03 on Days 0, 7, 14, 28, 42 and 56.
**Lokivetmab vs ciclosporin**	Moyaert et al., Vet Dermatol. 2017;28:593–e145. doi:10.1111/vde.12478<br><br>EMA Cytopoint EPAR	2017<br><br>EMA assessment adopted: 16 Feb 2017	**Design:** Multicenter, randomized, double-masked, double-dummy, positive-controlled noninferiority trial; 84 days, with an open-label lokivetmab extension for responders.<br>**Sample/allocation:** 274 client-owned dogs at 40 European practices; 142 lokivetmab and 132 ciclosporin (1:1).	**Case definition:** Chronic nonseasonal cAD diagnosed using published criteria; ≥6 months old; at least mild pruritus; CADESI-03 ≥60.<br>**Controls:** Food allergy, flea allergy, sarcoptic mange, ectoparasites, bacterial/fungal dermatitis and relevant metabolic disease excluded; flea prevention required; diet and immunotherapy stabilized.	**Washout:** Drug-specific intervals ranged from 1 week (oral antibacterials/topical anesthetics) to 6 weeks (long-acting corticosteroids/amantadine); oclacitinib, antihistamines and systemic azoles required 2 weeks.<br>**Concomitant therapy:** Potentially confounding therapies restricted;	**Co-primary endpoints:** Percentage reduction from baseline in owner PVAS and investigator CADESI-03 at Day 28; noninferiority margin 15%.<br>**Timing/rater:** PVAS on Days 0, 1, 2, 7, 14, 21 and 28, then monthly; CADESI-03 on Days 0, 14 and 28, then monthly. Remission categories and independent

Drug / comparison	Reference	Publication / assessment date	Study design and sample	Case definition, eligibility and disease controls	Washout and concomitant therapy	Endpoints, assessment timing and raters
					specified medications conditionally permitted.	response-to-treatment ratings were supportive.
**Ilunocitinib vs placebo**	Forster et al., Vet Dermatol. 2025;36:647–659. doi:10.1111/vde.13344<br><br>FDA FOI Summary, NADA 141-585 (AD Study ELA1900313)	Online: 29 Apr 2025<br><br>FDA approval: 19 Sep 2024	**Design:** Multicenter, randomized, double-masked, placebo-controlled field trial; 112 days.<br>**Sample/allocation:** 268 client-owned dogs at 25 US/Canadian clinics (dermatology and general practice); 181 ilunocitinib and 87 placebo (2:1, blocked within site). Treatment dispenser unmasked.	**Case definition:** Seasonal or nonseasonal cAD; ≥6/8 Favrot set 1 criteria; ≥12 months old; ≥3 kg; PVAS ≥6; CADESI-04 ≥25.<br>**Controls:** Dogs flea-free with continuous prevention; serious/systemic disease and selected confounding dermatoses excluded; stable concurrent conditions allowed.	**Washout:** Medication-specific intervals for JAK inhibitors, corticosteroids, ciclosporin, anti-IL-31 monoclonal antibodies, antimicrobials and local anesthetics; lokivetmab required 8 weeks.<br>**Concomitant therapy:** Systemic/topical or otic antimicrobials permitted after Day 28; stable noninterfering therapies allowed.	**Primary endpoint:** Composite Day 28 treatment success: ≥50% reduction from baseline in owner PVAS or investigator CADESI-04; early withdrawal for lack of efficacy counted as failure.<br>**Timing/rater:** PVAS daily during Week 1 and on Days 14, 28, 56, 84 and 112; CADESI-04 on Days 0, 14, 28, 56, 84 and 112. Owners completed PVAS before investigator consultation; independent response-to-treatment ratings were supportive.
**Ilunocitinib vs oclacitinib**	Forster et al., Vet Dermatol. 2025;36:165–176. doi:10.1111/vde.13319<br><br>EMA Zenrelia EPAR/public assessment report	Online: 6 Jan 2025<br><br>EU authorization: 24 Jul 2025; public assessment report: 6 Jan 2026	**Design:** Multicenter, randomized, blinded, active-controlled noninferiority trial; primary period to Day 56 with optional continuation to Day 112.<br>**Sample/allocation:** 338 client-owned dogs at 25 European clinics; 169 ilunocitinib and 169 oclacitinib (1:1, blocked). Owners masked; dispenser unmasked.	**Case definition:** cAD diagnosed using published recommendations; >12 months old; ≥3 kg; PVAS ≥6; CADESI-04 ≥35.<br>**Controls:** Food allergy, flea allergy, ectoparasites, bacterial/fungal dermatitis and relevant metabolic disease excluded; dogs flea-free with continuous control.	**Washout/concomitant therapy:** Protocol-defined washouts for interfering therapies; prohibited medication exposure within the preceding 90 days was an exclusion criterion. Stable, noninterfering concomitant medication was allowed.	**Co-primary endpoints:** Percentage reduction from baseline in owner PVAS and investigator CADESI-04 at Day 28; noninferiority margin 20% with multiplicity adjustment.<br>**Timing/rater:** Visits on Days 14, 28, 56, 84 and 112; owners maintained pruritus records. Response and remission outcomes were supportive.

Drug (product)	Regulatory authority	Document / date	Relationship to retained trial
Oclacitinib (Apoquel®)	FDA CVM (US)	Freedom of Information Summary, original NADA 141-345; approved 14 May 2013.	Identifies the AD effectiveness study and continuation study (A16/A17).
Oclacitinib (Apoquel®)	EMA/CVMP (EU)	Apoquel European public assessment report (EPAR); 2013.	Public EU assessment associated with the original authorization.
Lokivetmab (Cytopoint®)	EMA/CVMP (EU)	CVMP assessment report, EMEA/V/C/003939/0000; EMA/118401/2017; adopted 16 February 2017.	Identifies Moyaert et al. as the pivotal EU field efficacy trial; classifies the dose-titration study as supportive.
Ilunocitinib (Zenrelia™)	FDA CVM (US)	Freedom of Information Summary, original NADA 141-585; approved 19 September 2024.	Identifies the placebo-controlled AD effectiveness study ELA1900313.
Ilunocitinib (Zenrelia™)	EMA/CVMP (EU)	Zenrelia European public assessment report (EPAR); EU authorization 24 July 2025.	Associated with the pivotal active-comparator trial versus oclacitinib.

Scope and source matching. The table includes randomized, controlled field studies that specifically enrolled dogs with canine atopic dermatitis and public regulatory assessments that directly report or contextualize those studies. Journal articles and regulatory documents describing the same underlying trial are paired in one row. No USDA FOI-style public clinical-study summary was identified for Cytopoint; the EMA EPAR is therefore used as the public regulatory assessment.

Exclusions. Trials enrolling mixed allergic-dermatitis populations rather than an AD-defined population were excluded, including Apoquel Study 930/Cosgrove et al. (doi:10.1111/vde.12047), Gadeyne et al. (doi:10.1111/vde.12166), and Zenrelia Study ELA1900107/Forster et al. (doi:10.1111/vde.70009). Nonrandomized, retrospective and laboratory challenge studies were also excluded.

Abbreviations: AD, atopic dermatitis; cAD, canine atopic dermatitis; CADESI, Canine Atopic Dermatitis Extent and Severity Index; EPAR, European public assessment report; FDA, US Food and Drug Administration; FOI, Freedom of Information; JAK, Janus kinase; PVAS, pruritus visual analog scale. Sample sizes are numbers enrolled unless otherwise stated.

Regulatory-source note. No public USDA FOI-style clinical-study summary was identified
