# Supplementary material for: Canine atopic dermatitis clinical trials: study design and methodological considerations
Source: Front Vet Sci. 2026 Oct 9;13:1878714. doi: 10.3389/fvets.2026.1878714 (PMC13692925; doi:10.3389/fvets.2026.1878714)
Supplement: Supplementary file 2 [file Data_Sheet_2.PDF]

Supplementary Methods: Expert consultation

In spring 2022, the authors convened a single two-hour virtual roundtable attended concurrently by all seven experts acknowledged in the manuscript. The consultation was originally conducted to inform development of a pilot study protocol evaluating OKV-1001 in dogs with moderate-to-severe and/or refractory canine AD; perspectives arising from the discussion were subsequently used to contextualize methodological trade-offs considered in this narrative review. Participants were selected based on their published expertise and/or clinical trial experience in canine AD. Participation was voluntary and uncompensated. At the time of the consultation, none of the experts had a relevant financial relationship with Okava Pharmaceuticals.

The discussion was guided by a slide presentation and draft protocol prepared in advance. Topics identified in advance included the balance between internal validity and generalizability; target population and disease severity; case definition and eligibility criteria; food allergy, ectoparasite, and infection control; antimicrobial and topical-therapy restrictions; washout periods; glucocorticoid and other concomitant therapy; endpoint selection and assessment timing; and recruitment feasibility. No structured written feedback or survey was collected; individual participants were subsequently contacted by telephone or email on an ad hoc basis to clarify specific points. The authors documented and synthesized the discussion and used it to inform the manuscript sections addressing case definition and eligibility, infection and ectoparasite control, washout and concomitant therapies, endpoint selection and timing, and trial feasibility and generalizability.

The consultation was advisory rather than a formal consensus process. No voting, prespecified consensus threshold, or formal procedure for adjudicating disagreement was used. Divergent perspectives were considered qualitatively by the authors, who retained responsibility for the manuscript’s interpretation and final content. The experts did not review or approve the final manuscript.
